# Formation of a di­iron–(μ-η^1^:η^1^-CN) com­plex from aceto­nitrile solution

**DOI:** 10.1107/S2053229624007058

**Published:** 2024-08-08

**Authors:** Tim P. Schlachta, Michael J. Sauer, Leon F. Richter, Fritz E. Kühn

**Affiliations:** a Technical University of Munich, School of Natural Sciences, Department of Chemistry and Catalysis Research Center, Molecular Catalysis, Lichtenbergstrasse 4, 85748 Garching, Germany; University of Monash, Australia

**Keywords:** organometallic iron(II) complex, acetonitrile cleavage, N-heterocyclic carbene, NHC, crystal structure, cyanation

## Abstract

The activation of C—C bonds by transition-metal com­plexes is of continuing inter­est and aceto­nitrile (MeCN) has attracted attention as a cyanide source with com­paratively low toxicity for organic cyanation reactions. A di­iron end-on μ-η^1^:η^1^-CN-bridged com­plex was obtained from a crystallization experiment of an open-chain iron–NHC com­plex, namely, μ-cyanido-κ^2^*C*:*N*-bis­{[(aceto­nitrile-κ*N*)[3,3′-bis­(pyridin-2-yl)-1,1′-(methyl­idene)bis­(benzimidazol-2-yl­idene)]iron(II)} tris­(hexa­fluoro­phos­phate), [Fe_2_(CN)(C_2_H_3_N)_2_(C_25_H_18_N_6_)_2_](PF_6_)_3_. The cyanide appears to originate from the MeCN solvent by C—C bond cleavage or through carbon–hy­dro­gen oxidation.

## Abstract 2

A di­iron end-on μ_2_-η^1^:η^1^-CN-bridged com­plex is obtained from a crystallization experiment of an open-chain iron–NHC com­plex. The cyanide appears to originate from the aceto­nitrile (MeCN) solvent by C—C bond cleavage or through carbon–hy­dro­gen oxidation.

## Introduction

The first iron–NHC (N-heterocyclic carbene) com­plex was developed by Öfele in 1969 (Öfele, 1969 ▸). However, it has taken many years for iron–NHC com­plexes to attract the attention of a wider audience of chemists, but, especially in the last decade, there has been a sharp increase in related publications (Riener *et al.*, 2014 ▸). The open-chain iron–pyridine-NHC com­plex bis­(aceto­nitrile-κ*N*)[3,3′-bis­(pyridin-2-yl)-1,1′-(methyl­idene)bis­(benzimidazol-2-yl­idene)]iron(II) bis­(hexa­fluoro­phos­phate), **1** (Scheme 1), can be successfully employed in homogeneous epoxidation catalysis (Schlachta *et al.*, 2024 ▸). In the present work, a di­iron end-on μ-η^1^:η^1^-CN-bridged com­plex, **2** (Scheme 2), is formed from a solution of **1** in deu­ter­ated aceto­nitrile. The activation of C—C bonds by tran­si­tion-metal com­plexes is of continuing inter­est and MeCN has attracted attention as a cyanide source with com­paratively low toxicity for organic cyanation reactions (Ahmad *et al.*, 2020 ▸; Lu *et al.*, 2004 ▸; Spentzos *et al.*, 2020 ▸; Grirrane *et al.*, 2016 ▸).

## Experimental

### General procedures and analytical methods

Complex **1** was synthesized according to a literature method (Schlachta *et al.*, 2024 ▸). Solvents were purified, dried and de­gassed using standard methods (Armarego, 2017 ▸) or received from a solvent purification system by M. Braun. All other chemicals were obtained from commercial suppliers and were used without further purification. NMR spectra were recorded on a Bruker Avance Ultrashield AV400 (400.13 MHz for ^1^H NMR and 100.53 MHz for ^13^C NMR). The chemical shifts are given in δ values in ppm (parts per million) relative to TMS (tetra­methyl­silane) and are reported relative to the residual deuterated solvent signal (Fulmer *et al.*, 2010 ▸). Electrospray ionization mass spectrometry (ESI–MS) data were measured on a Thermo Fisher Ultimate 3000. FT–IR measurements were conducted on a PerkinElmer Frontier FT–IR spec­trom­eter (ATR). The ‘inVia Reflex Raman System’ com­prises a research grade optical microscope [Leica DM2700M, Magnification 5×, 20× and 50× (in this case, 50× was used)] coupled to a high-performance Raman spectrometer (Renishaw). A 633 nm wavelength laser was used (Renishaw RL633 Class 3B).
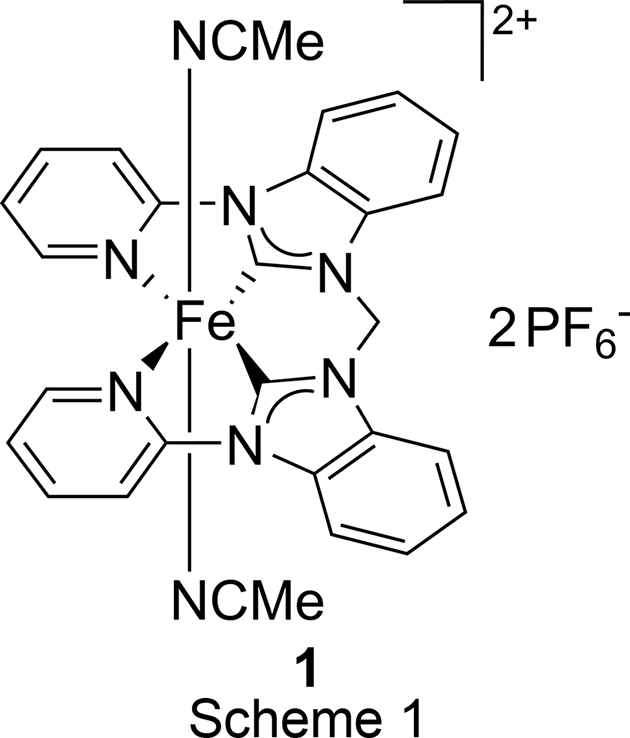

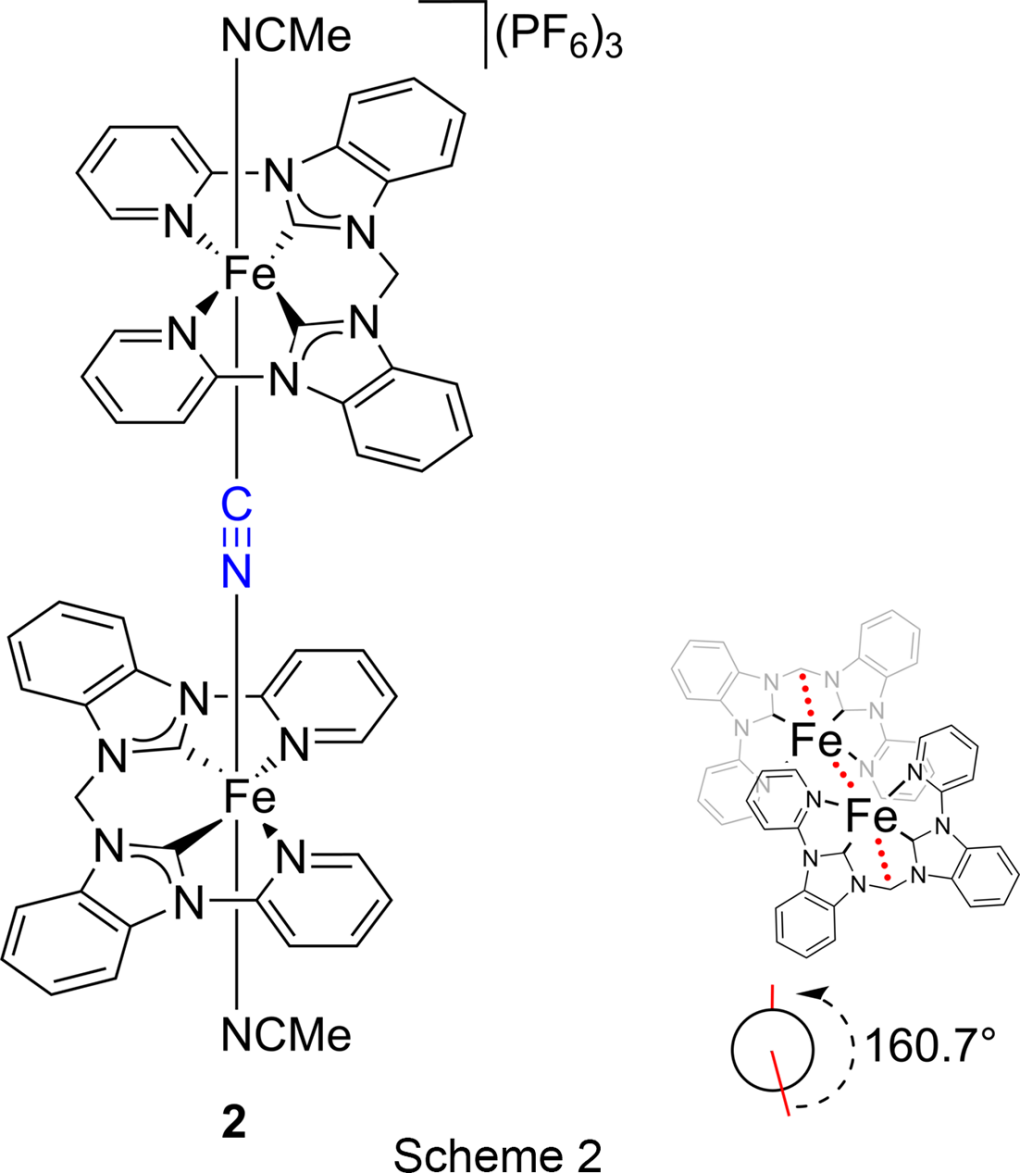


### Crystallization of 2

Single crystals of **2** suitable for X-ray diffraction were obtained by slow evaporation of a solution of **1** in CD_3_CN over a period of six months at room temperature under an ambient atmosphere near a window with sunlight (see supporting information).

A solution of **1** (around 1–2 mg) in CD_3_CN (around 0.4 ml, dry and degassed) from an NMR tube (see supporting information) was placed in a 10 ml vial under an ambient atmosphere. A human hair was fixed with adhesive tape to the inside of the vial, reaching into the solution. Heterogeneous nu­cle­ation occurs more frequently than homogeneous nucleation (Sear, 2014 ▸; Pruppacher & Klett, 1997 ▸) and human hair has been used for the growth of nanoparticles or as catalyst-support material (Deng *et al.*, 2016 ▸; Liu *et al.*, 2015 ▸; Haveli *et al.*, 2012 ▸; Walter *et al.*, 2006 ▸). The vial was closed and the cap was punctured with a cannula. The vial was left for six months at room temperature under ambient conditions near a window with sunlight, allowing the solvent to evaporate slowly. Orange crystals suitable for SC-XRD analysis were obtained.

### Refinement

Crystal data, data collection and structure refinement details are summarized in Table 1 ▸. H atoms could not be located in difference Fourier maps and were calculated in ideal positions (riding model), with C—H = 0.98 Å and *U*_iso_(H) = 1.5*U*_eq_(C) for CH_3_ groups, C—H = 0.99 Å and *U*_iso_(H) = 1.2*U*_eq_(C) for CH_2_ groups, and C—H = 0.95 Å and *U*_iso_(H) = 1.2*U*_eq_(C) for CH groups. Split-layer position refinement was used for atoms P2, F7, F8, F9, F10, F11 and F12 (PF_6_^−^ anion), as well as N8 and C28 (bridging cyanide). Restraints were applied to atoms N8 and C28 to ensure reasonable ellipsoids. CD_3_ has been modelled as CH_3_ as there is no appreciable difference in SC-XRD.

## Results and discussion

**Figure 1:**
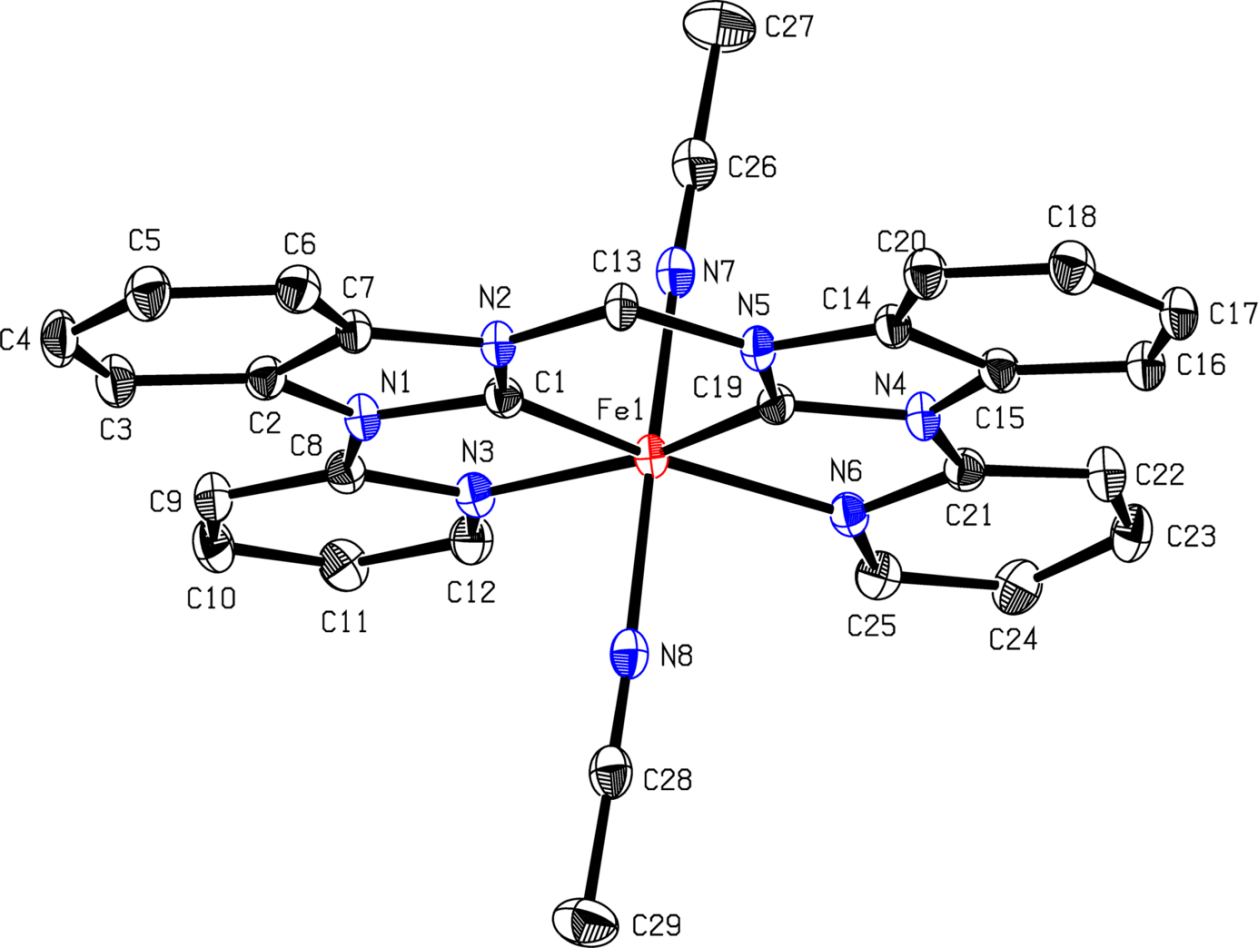
The mol­ecular structure of **1**. H atoms and hexa­fluoro­phos­phate anions have been omitted for clarity. Displacement ellipsoids are shown at the 50% probability level (Schlachta *et al.*, 2024 ▸).

**Figure 2:**
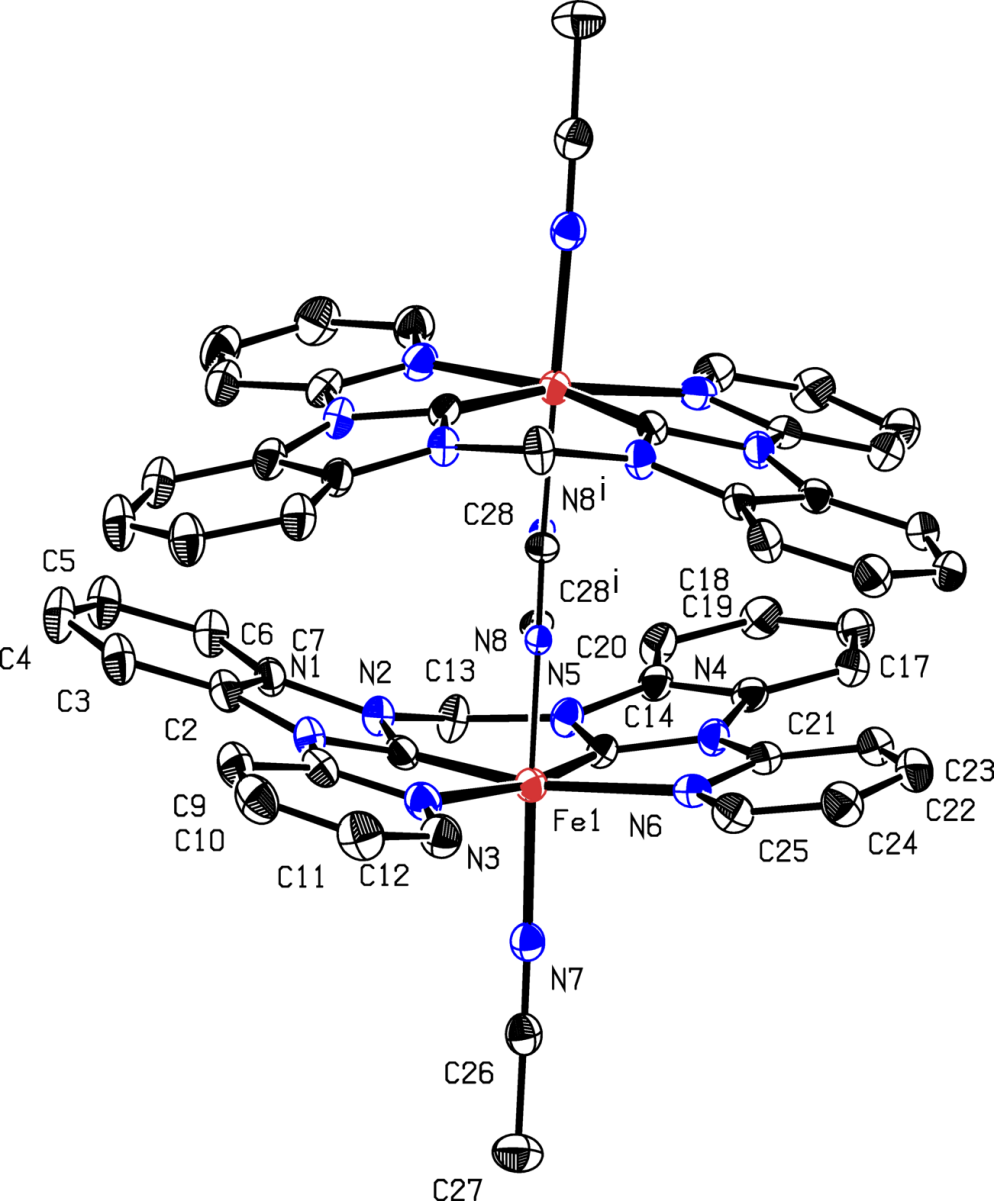
The mol­ecular structure of **2**. H atoms and hexa­fluoro­phos­phate anions have been omitted for clarity. Displacement ellipsoids are shown at the 50% probability level.

When a solution of **1** (Scheme 1 and Fig. 1 ▸) in CD_3_CN was evaporated slowly over a period of six months under ambient conditions, a di­iron end-on μ-η^1^:η^1^-CN-bridged com­plex, [(MeCN)(NHC)Fe]_2_(μ-η^1^:η^1^-CN)(PF_6_)_3_ (**2**) (Scheme 2 and Fig. 2 ▸), was obtained, as determined by X-ray diffraction. The two iron centres are bridged by a cyanide anion, hence three PF_6_^−^ anions are present in the crystal structure. Under similar conditions, *i.e.* MeCN solution, room temperature and air, a dinuclear Cu^II^ cryptate has been found to form a μ-η^1^:η^1^-CN-bridged com­plex by C—C bond cleavage of MeCN (Lu *et al.*, 2004 ▸). A possible mechanism involving the activation of the *sp*-hybridized C atom of MeCN, bound to one Cu atom (MeCN—Cu), by the second Cu centre has been suggested. The increased electrophilicity of the methyl group would allow cleavage by H_2_O to form MeOH and the cyanide-bridged com­pound (Lu *et al.*, 2004 ▸; Ahmad *et al.*, 2020 ▸). Another possible mechanism for the formation of **2** might be the carbon–hy­dro­gen oxidation of MeCN by iron com­plex **1** to form glycolo­nitrile, as observed previously for an iron(III) tetra­carbene com­plex, and subsequent release of cyanide upon decay of glycolo­nitrile (Knapp *et al.*, 2012 ▸; Dyckhoff *et al.*, 2021 ▸; Lewis, 2008 ▸). Due to the stronger Me—CN bond (122 kcal mol^−1^) com­pared to the H—CH_2_CN bond (93 kcal mol^−1^) (Spentzos *et al.*, 2020 ▸; Blanksby & Ellison, 2003 ▸; Goebbert *et al.*, 2010 ▸; Miscione & Bottoni, 2014 ▸), the carbon–hy­dro­gen oxidation of MeCN seems to be more likely the origin of cyanide in this case. However, C—C bond cleavage of MeCN by UV irradiation is known (Grirrane *et al.*, 2016 ▸) and, given the fact that the crystallization setup with **1** was also accessible for sunlight during the extensive period of six months, C—C bond cleavage of MeCN cannot be excluded.

The crystal structure of **2** reveals strongly bent equatorial NHC ligands. This finding is in stark contrast to **1**, where the NHC ligand is largely planar (Fig. 1 ▸). This sandwich-like structure encapsulates the cyanide ion and is indicative of some noncovalent inter­actions between the equatorial ligands, likely contributing to the stability of **2**. Inter­estingly, the py­ri­dine units are bent less towards the centre com­pared to the NHC units, forming a Z-shape or diamond-shape, depending on the viewing angle of **2**. The Fe—N—C angle is slightly bent (Table 2 ▸) in a *trans* fashion, resulting in a ‘zigzag’ vertical axis. Another inter­esting finding is the rotation of the NHC ligands towards each other in an *anti* conformation, resulting in a dihedral angle (CH_2_—Fe—Fe—CH_2_) of 160.7° (Scheme 2). The crystal structure can in principle also be solved as the di­iron–(μ-η^1^:η^1^-N_2_) com­plex (Fig. 2 ▸), which is why we refrain from a detailed structural discussion at this point. However, there are several arguments against a di­iron–(μ-η^1^:η^1^-N_2_) com­plex:

(i) The main argument against a di­iron–(μ-η^1^:η^1^-N_2_) com­plex is the fact that the crystal structure contains three counter-ions. As the crystallization was performed with **1** containing an iron(II) centre, bridging two Fe^II^ atoms with a neutral N_2_ ligand should lead to the presence of four counter-ions. Otherwise, three counter-ions would indicate that a redox process has occurred during the formation of **2**, but the nature of a hypothetical reducing agent and the location of reduction are highly speculative. The main com­ponents of the crystallization experiment were **1** and CD_3_CN, as well as un­reacted ligand precursor as a minor impurity (see sup­porting information). In a cyclic voltammetry study of **1**, the first reduction event occurred at −1.78 V (*versus* Fc/Fc^+^). A pre­limi­nary experiment measuring **1** in cyclic voltammetry under an N_2_ atmosphere did not show significant redox processes or electric current. Considering all these facts, the involvement of a redox process appears to be quite implausible.

(ii) Di­nitro­gen is a weak σ-donor and a weak π-acceptor, and substitution of the N_2_ ligand with CO or nitriles like MeCN is often observed (Crossland & Tyler, 2010 ▸; Sunada *et al.*, 2013 ▸). A di­iron–(μ-η^1^:η^1^-N_2_) version of **2** would be very surprising in this context, since one axial MeCN ligand coordinates with one iron centre each, the crystallization of **2** occurred in (deuterated) MeCN as solvent and the previous occupation of both axial coordination sites by MeCN in **1**. The stability of **2** under air is also inter­esting, which would be rather uncommon for a di­iron–(μ-η^1^:η^1^-N_2_) com­plex (Crossland & Tyler, 2010 ▸; Takeshita *et al.*, 2018 ▸; Saouma *et al.*, 2011 ▸; Regenauer *et al.*, 2022 ▸) and an affinity for N_2_ over O_2_ would be very unusual considering other Fe com­pounds tending to form di­iron–μ-oxido species (Schlachta & Kühn, 2023 ▸; Schlachta *et al.*, 2021 ▸).

(iii) A di­iron–(μ-η^1^:η^1^-N_2_) com­plex should show a distinctive *v*_NN_ absorption band in Raman spectroscopy and be IR inactive due to the centrosymmetric structure (Suess & Peters, 2013 ▸; McWilliams *et al.*, 2018 ▸; Gu *et al.*, 2018 ▸). No *v*_NN_ band was detected in the crude material either by IR or Raman spectroscopy. However, no pronounced *v*_CN_ stretch could be observed either and, inter­estingly, com­plex **1** also does not show a characteristic *v*_CN_ band in IR, contrary to similar com­plexes (Raba *et al.*, 2012 ▸), but signals attributable to axial MeCN are visible in the Raman spectrum (see supporting information).

## Conclusion

A di­iron end-on μ-η^1^:η^1^-CN-bridged com­plex, **2**, was obtained from a crystallization experiment with an open-chain iron NHC com­plex **1**. The cyanide presumably originates from the MeCN solvent by C—C bond cleavage or through carbon–hy­dro­gen oxidation. The strongly bent NHC ligands are positioned in an *anti* conformation.

## Supplementary Material

Crystal structure: contains datablock(s) I, global. DOI: 10.1107/S2053229624007058/jx3087sup1.cif

Structure factors: contains datablock(s) I. DOI: 10.1107/S2053229624007058/jx3087Isup2.hkl

Supporting information. DOI: 10.1107/S2053229624007058/jx3087sup3.pdf

CCDC reference: 2326821

## Figures and Tables

**Table 1:** Experimental details

Crystal data	
Chemical formula	[Fe_2_(CN)(C_2_D_3_N)_2_(C_25_H_18_N_6_)_2_](PF_6_)_3_
*M*_r_	1465.67
Crystal system, space group	Monoclinic, *C*2/*c*
Temperature (K)	100
*a*, *b*, *c* (Å)	25.562 (2), 17.0373 (15), 14.8998 (12)
β (°)	112.112 (3)
*V* (Å^3^)	6011.8 (9)
*Z*	4
Radiation type	Mo *K*α
μ (mm^−1^)	0.67
Crystal size (mm)	0.13 × 0.05 × 0.04

Data collection	
Diffractometer	Bruker D8 Venture
Absorption correction	Multi-scan (*SADABS*; Bruker, 2016 ▸)
*T*_min_, *T*_max_	0.708, 0.745
No. of measured, independent and observed [*I* > 2σ(*I*)] reflections	89835, 5310, 4621
*R*_int_	0.051
(sin θ/λ)_max_ (Å^−1^)	0.595

Refinement	
*R*[*F*^2^ > 2σ(*F*^2^)], *wR*(*F*^2^), *S*	0.031, 0.083, 1.11
No. of reflections	5310
No. of parameters	461
No. of restraints	9
H-atom treatment	H-atom parameters constrained
Δρ_max_, Δρ_min_ (e Å^−3^)	0.43, −0.27

Computer programs: *APEX4* (Bruker, 2021 ▸), *SAINT* (Bruker, 2019 ▸), *SHELXT* (Sheldrick, 2015*a* ▸), *SHELXL2019* (Sheldrick, 2015*b* ▸), *PLATON* (Spek, 2020 ▸) and *enCIFer* (Allen *et al.*, 2004 ▸).

**Table 2:** Selected geometric parameters (Å, °)

Fe1—N3	2.0703 (16)	Fe1—N8	1.83 (4)
Fe1—N6	2.0754 (17)	Fe1—C1	1.824 (2)
Fe1—N7	1.9336 (17)	Fe1—C14	1.824 (2)
Fe1—C28^i^	1.98 (5)	N8—C28	1.128 (12)
			
N7—Fe1—N8	173.3 (10)	Fe1—N8—C28	171.1 (7)
Fe1^i^—C28—N8	177 (4)	N3—Fe1—C14	167.26 (8)

Symmetry code: (i) 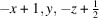.

## References

[bb1] Ahmad, M. S., Pulidindi, I. N. & Li, C. (2020). *New J. Chem.* **44**, 17177–17197.

[bb2] Allen, F. H., Johnson, O., Shields, G. P., Smith, B. R. & Towler, M. (2004). *J. Appl. Cryst.* **37**, 335–338.

[bb3] Armarego, W. L. (2017). In *Purification of laboratory chemicals*. London: Butterworth–Heinemann.

[bb4] Blanksby, S. J. & Ellison, G. B. (2003). *Acc. Chem. Res.* **36**, 255–263. 10.1021/ar020230d 12693923

[bb5] Bruker (2016). *SADABS*. Bruker AXS Inc., Madison, Wisconsin, USA.

[bb6] Bruker (2019). *SAINT*. Bruker AXS Inc., Madison, Wisconsin, USA.

[bb7] Bruker (2021). *APEX4*. Bruker AXS Inc., Madison, Wisconsin, USA.

[bb8] Crossland, J. L. & Tyler, D. R. (2010). *Coord. Chem. Rev.* **254**, 1883–1894.

[bb9] Deng, D., Gopiraman, M., Kim, S. H., Chung, I.-M. & Kim, I. S. (2016). *ACS Sustainable Chem. Eng.* **4**, 5409–5414.

[bb10] Dyckhoff, F., Schlagintweit, J. F., Bernd, M. A., Jakob, C. H. G., Schlachta, T. P., Hofmann, B. J., Reich, R. M. & Kühn, F. E. (2021). *Catal. Sci. Technol.* **11**, 795–799.

[bb11] Fulmer, G. R., Miller, A. J. M., Sherden, N. H., Gottlieb, H. E., Nudelman, A., Stoltz, B. M., Bercaw, J. E. & Goldberg, K. I. (2010). *Organometallics*, **29**, 2176–2179.

[bb12] Goebbert, D. J., Velarde, L., Khuseynov, D. & Sanov, A. (2010). *J. Phys. Chem. Lett.* **1**, 792–795.

[bb13] Grirrane, A., Álvarez, E., Albero, J., García, H. & Corma, A. (2016). *Dalton Trans.* **45**, 5444–5450. 10.1039/c6dt00370b 26959701

[bb14] Gu, N. X., Oyala, P. H. & Peters, J. C. (2018). *J. Am. Chem. Soc.* **140**, 6374–6382. 10.1021/jacs.8b02603 PMC6592702 29684269

[bb15] Haveli, S. D., Walter, P., Patriarche, G., Ayache, J., Castaing, J., Van Elslande, E., Tsoucaris, G., Wang, P.-A. & Kagan, H. B. (2012). *Nano Lett.* **12**, 6212–6217. 10.1021/nl303107w 23126235

[bb16] Knapp, S. M. M., Sherbow, T. J., Juliette, J. J. & Tyler, D. R. (2012). *Organometallics*, **31**, 2941–2944.

[bb17] Lewis, R. J. Sr (2008). In *Hazardous Chemicals Desk Reference*, 6th ed. Chichester: John Wiley & Sons.

[bb18] Liu, X., Zhou, W., Yang, L., Li, L., Zhang, Z., Ke, Y. & Chen, S. (2015). *J. Mater. Chem. A*, **3**, 8840–8846.

[bb19] Lu, T., Zhuang, X., Li, Y. & Chen, S. (2004). *J. Am. Chem. Soc.* **126**, 4760–4761. 10.1021/ja031874z 15080663

[bb20] McWilliams, S. F., Bunting, P. C., Kathiresan, V., Mercado, B. Q., Hoffman, B. M., Long, J. R. & Holland, P. L. (2018). *Chem. Commun.* **54**, 13339–13342. 10.1039/c8cc07294a PMC6258315 30403226

[bb21] Miscione, G. P. & Bottoni, A. (2014). *Organometallics*, **33**, 4173–4182.

[bb22] Öfele, K. (1969). *Angew. Chem. Int. Ed. Engl.* **8**, 916–917.

[bb23] Pruppacher, H. R. & Klett, J. D. (1997). In *Microphysics of Clouds and Precipitation*. Dordrecht: Springer.

[bb24] Raba, A., Cokoja, M., Ewald, S., Riener, K., Herdtweck, E., Pöthig, A., Herrmann, W. A. & Kühn, F. E. (2012). *Organometallics*, **31**, 2793–2800.

[bb25] Regenauer, N. I., Wadepohl, H. & Roşca, D.-A. (2022). *Inorg. Chem.* **61**, 7426–7435. 10.1021/acs.inorgchem.2c00459 35508073

[bb26] Riener, K., Haslinger, S., Raba, A., Högerl, M. P., Cokoja, M., Herrmann, W. A. & Kühn, F. E. (2014). *Chem. Rev.* **114**, 5215–5272. 10.1021/cr4006439 24655079

[bb27] Saouma, C. T., Moore, C. E., Rheingold, A. L. & Peters, J. C. (2011). *Inorg. Chem.* **50**, 11285–11287. 10.1021/ic2016066 PMC3215824 22004139

[bb28] Schlachta, T. P., Anneser, M. R., Schlagintweit, J. F., Jakob, C. H. G., Hintermeier, C., Böth, A. D., Haslinger, S., Reich, R. M. & Kühn, F. E. (2021). *Chem. Commun.* **57**, 6644–6647. 10.1039/d1cc02027g 34126626

[bb29] Schlachta, T. P. & Kühn, F. E. (2023). *Chem. Soc. Rev.* **52**, 2238–2277. 10.1039/d2cs01064j 36852959

[bb30] Schlachta, T. P., Zámbó, G. G., Sauer, M. J., Rüter, I. & Kühn, F. E. (2024). Submitted. 10.1002/open.202400071 PMC11625922 39318071

[bb31] Sear, R. P. (2014). *CrystEngComm*, **16**, 6506–6522.

[bb32] Sheldrick, G. M. (2015*a*). *Acta Cryst.* A**71**, 3–8.

[bb33] Sheldrick, G. M. (2015*b*). *Acta Cryst.* C**71**, 3–8.

[bb34] Spek, A. L. (2020). *Acta Cryst.* E**76**, 1–11. 10.1107/S2056989019016244 PMC6944088 31921444

[bb35] Spentzos, A. Z., Gau, M. R., Carroll, P. J. & Tomson, N. C. (2020). *Chem. Commun.* **56**, 9675–9678. 10.1039/d0cc03521a PMC7442599 32696777

[bb36] Suess, D. L. M. & Peters, J. C. (2013). *J. Am. Chem. Soc.* **135**, 4938–4941. 10.1021/ja400836u PMC3770781 23472709

[bb37] Sunada, Y., Imaoka, T. & Nagashima, H. (2013). *Organometallics*, **32**, 2112–2120.

[bb38] Takeshita, T., Sato, K. & Nakajima, Y. (2018). *Dalton Trans.* **47**, 17004–17010. 10.1039/c8dt04168g 30460962

[bb39] Walter, P., Welcomme, E., Hallégot, P., Zaluzec, N. J., Deeb, C., Castaing, J., Veyssière, P., Bréniaux, R., Lévêque, J.-L. & Tsoucaris, G. (2006). *Nano Lett.* **6**, 2215–2219. 10.1021/nl061493u 17034086

