# Supplementary material for: Formation of a di­iron–(μ-η1:η1-CN) com­plex from aceto­nitrile solution
Source: Acta Crystallogr C Struct Chem. 2024 Aug 8;80(Pt 9):534–7. doi: 10.1107/S2053229624007058 (PMC11370999; doi:10.1107/S2053229624007058)
Supplement: Supplementary file 3 [file c-80-00534-sup3.pdf]

Supporting Information (SI)

Formation of a diiron-(μ-η^1:η^1-CN) complex from acetonitrile solution

Tim P. Schlachta, Michael J. Sauer, Leon F. Richter, Fritz E. Kühn*

Technical University of Munich, School of Natural Sciences, Department of Chemistry and Catalysis Research Center, Molecular Catalysis, Lichtenbergstraße 4, 85748 Garching, Germany. E-mail: fritz.kuehn@ch.tum.de; Phone (secretary's office): +49 (0)89 289 13477

[*] Corresponding Author

Table of contents

1. General procedures and analytical methods 2
2. Crystallographic data 3
3. IR Spectroscopy 5
4. Raman spectroscopy 8
5. References SI 11

1. General procedures and analytical methods

Complex **1** was synthesized according to the literature. Solvents were purified, dried and degassed using standard methods(Armarego, 2017) or received from a solvent purification system by M. Braun. All other chemicals were obtained from commercial suppliers and were used without further purification. NMR spectra were recorded on a Bruker Avance Ultrashield AV400 (^{1}H NMR, 400.13 MHz; ^{13}C NMR, 100.53 MHz). The chemical shifts are given in δ values in ppm (parts per million) relative to TMS (tetramethylsilane) and are reported relative to the residual deuterated solvent signal.(Fulmer *et al.*, 2010) Electrospray ionization mass spectrometry (ESI-MS) data were measured on a Thermo Fisher Ultimate 3000. FT-IR measurements were conducted on a *PerkinElmer* Frontier FT-IR spectrometer (ATR). The "inVia Reflex Raman System" comprises a research grade optical microscope (Leica DM2700M, Magnification 5x, 20x, 50x (in this case 50x was used)) coupled to a high performance Raman spectrometer (Renishaw). The 633nm wavelength Laser was used: RL633, Class 3B, Company: Renishaw.

CCDC 2326821 (complex **2**) contains the supplementary crystallographic data for this paper. This data can be obtained free of charge *via* www.ccdc.cam.ac.uk/data_request/cif, or by emailing data_request@ccdc.cam.ac.uk, or by contacting The Cambridge Crystallographic Data Centre, 12 Union Road, Cambridge CB2 1EZ, UK; fax: +44 1223 336033.

2. Crystallographic data

Crystallographic data of 2

Single crystals suitable for X-ray diffraction were obtained by slow evaporation of a solution of **1** in CD_3CN over 6 months at r.t. under ambient atmosphere near a window with sunlight (see Figure 1 SI):

A solution of **1** (around 1-2 mg) in CD_3CN (around 0.4 mL, dry and degassed) from an NMR tube (Figure 2 SI, Figure 3 SI) was placed in a 10 mL vial under ambient atmosphere. A human hair from the first author was fixed with adhesive tape to the inside of the vial, reaching into the solution. Heterogeneous nucleation occurs more frequently than homogeneous nucleation(Sear, 2014, Pruppacher & Klett, 1997) and human hair has been used for growth of nanoparticles or as catalyst support material.(Deng *et al.*, 2016, Liu *et al.*, 2015, Haveli *et al.*, 2012, Walter *et al.*, 2006) The vial was closed, and the cap was punctured with a cannula. The vial was left 6 months at r.t. under ambient conditions near a window with sunlight, allowing the solvent to slowly evaporate and orange crystals suitable for SC-XRD were obtained.

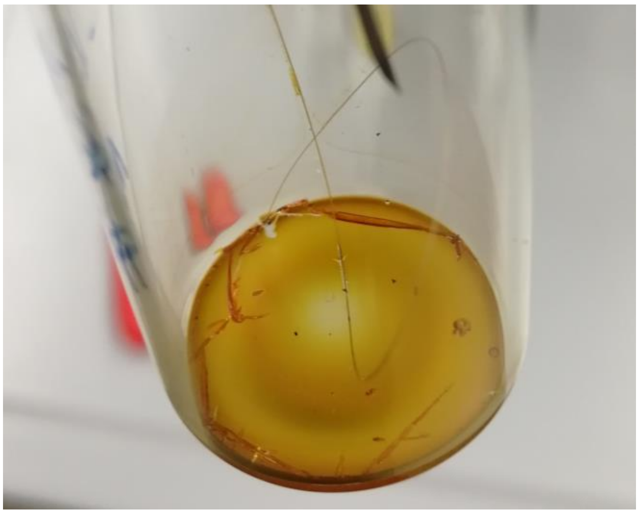

Figure 1 SI. Crystallization setup of **2**.

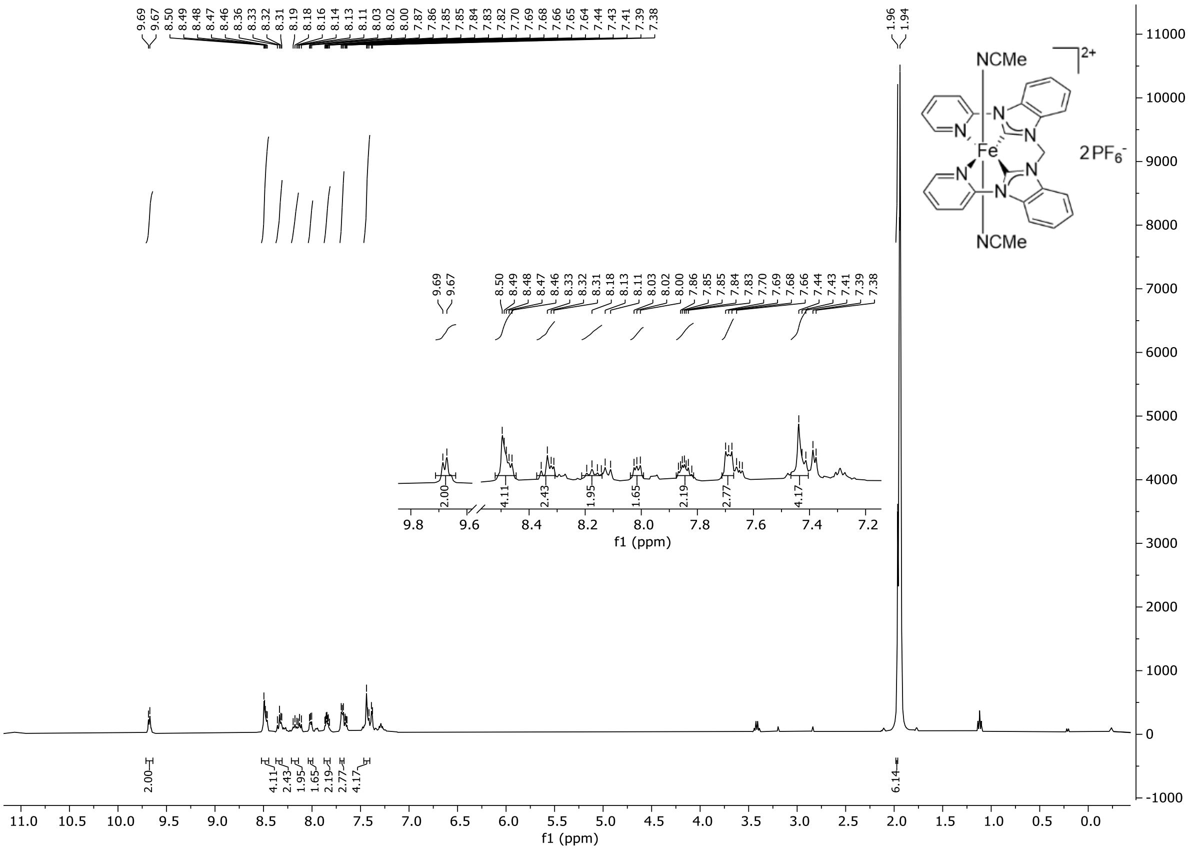

Figure 2 SI. ^{1}H NMR spectrum of **1** in CD_3CN before crystallization. Unreacted NHC ligand precursor is visible as impurity.

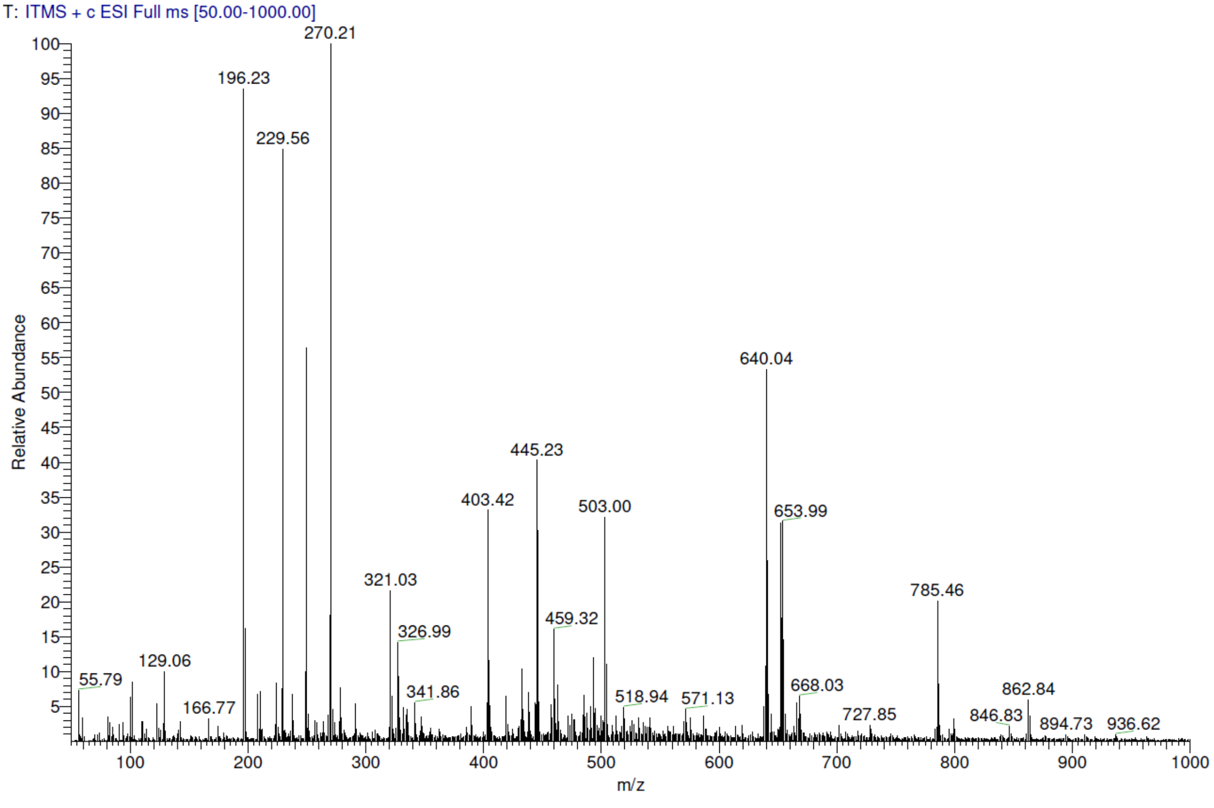

Figure 3 SI. ESI-MS spectrum of the batch of **1** before crystallization. Unreacted NHC ligand precursor is still present as impurity.

3. IR Spectroscopy

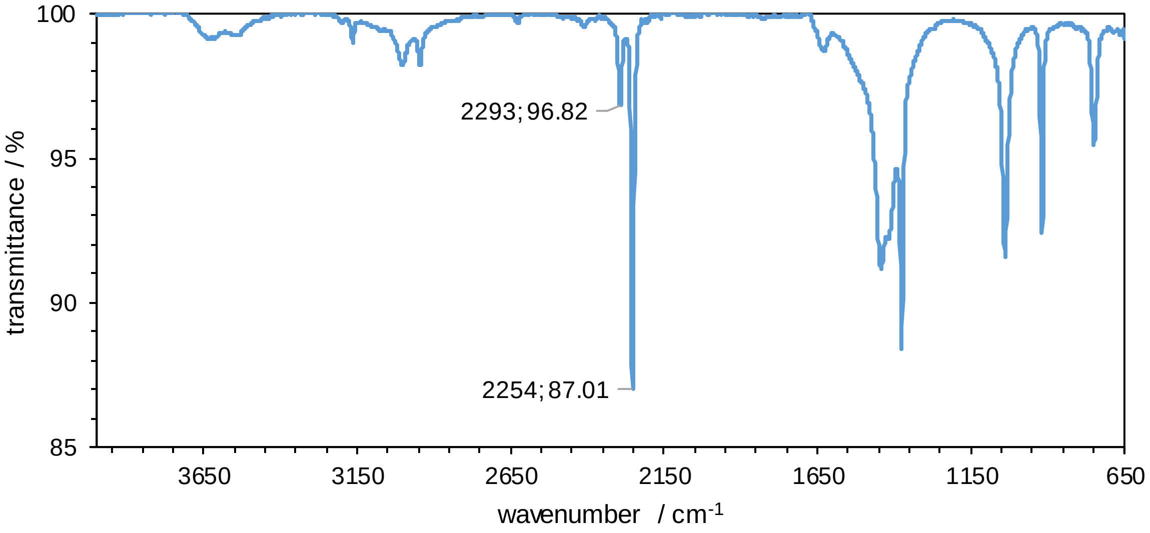

Figure 4 SI. FT-IR spectrum of MeCN. The values are in accordance to the literature.(Dereka *et al.*, 2022)

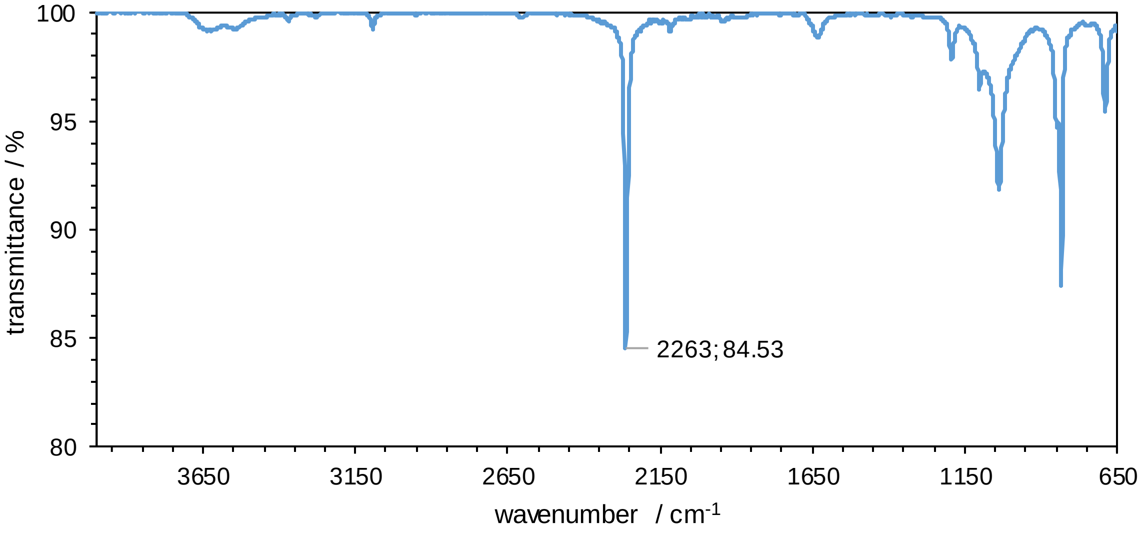

Figure 5 SI. FT-IR spectrum of CD_3CN. The values are in accordance to the literature.(Dereka *et al.*, 2022)

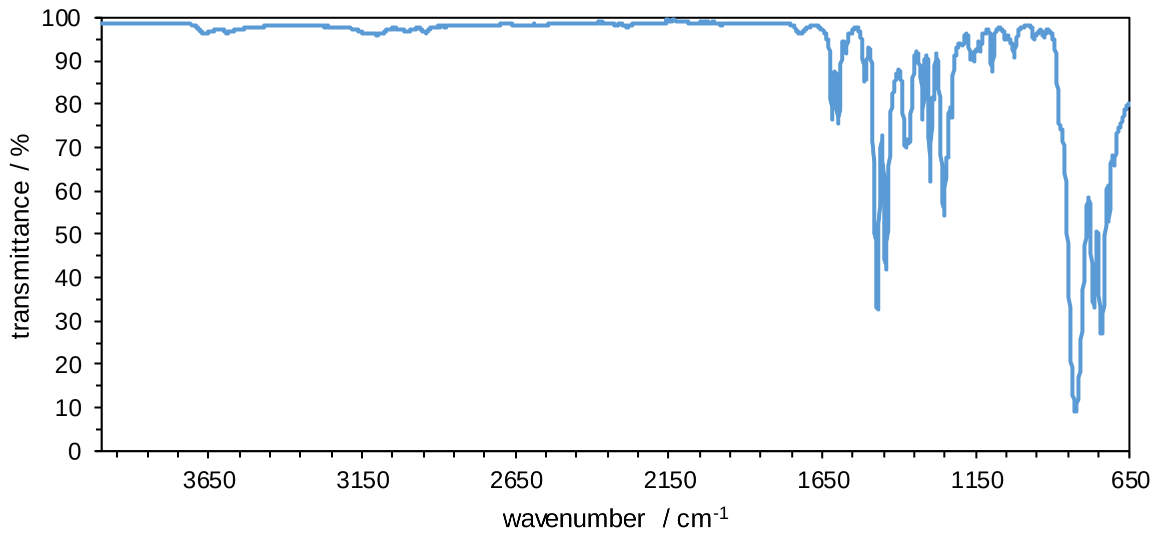

Figure 6 SI. FT-IR spectrum of **1**. No CN band is visible.

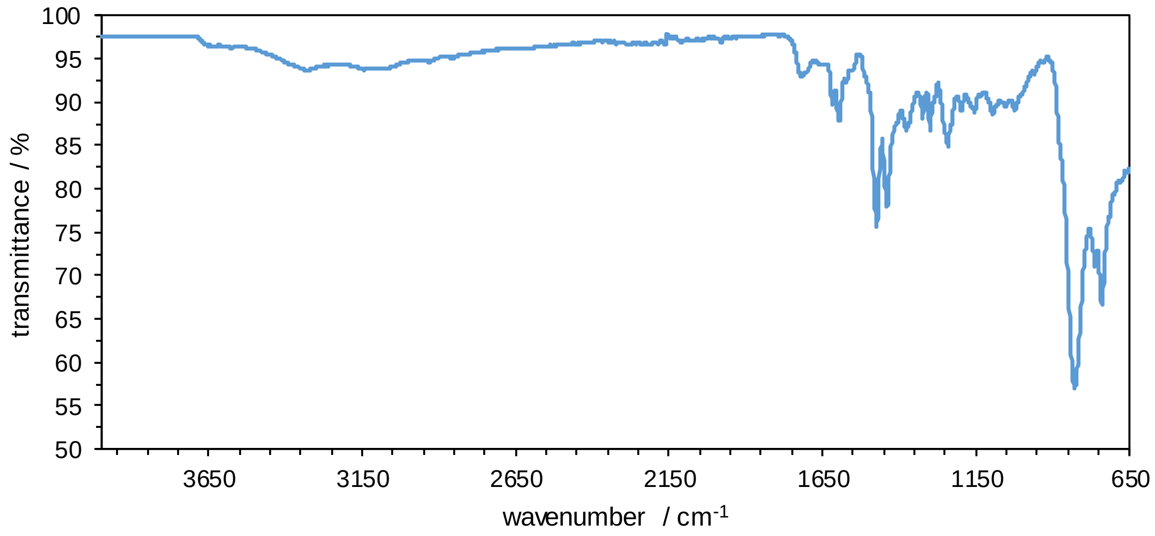

Figure 7 SI. FT-IR spectrum of crude material of the crystallization batch of **2**. No CN band is visible.

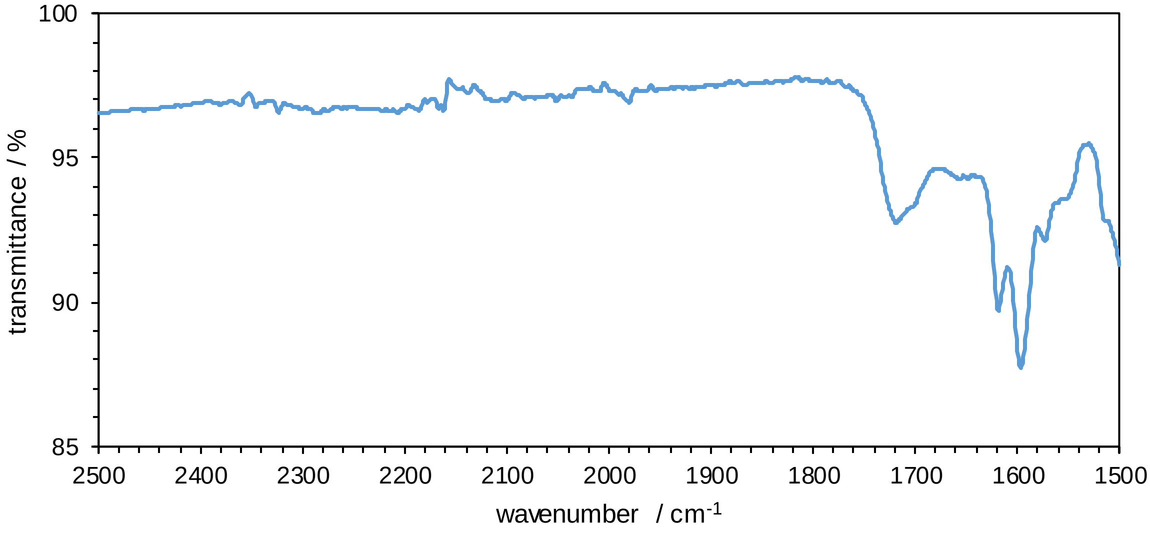

Figure 8 SI. Close up of the FT-IR spectrum of crude material of the crystallization batch of **2**. No CN band is visible.

4. Raman spectroscopy

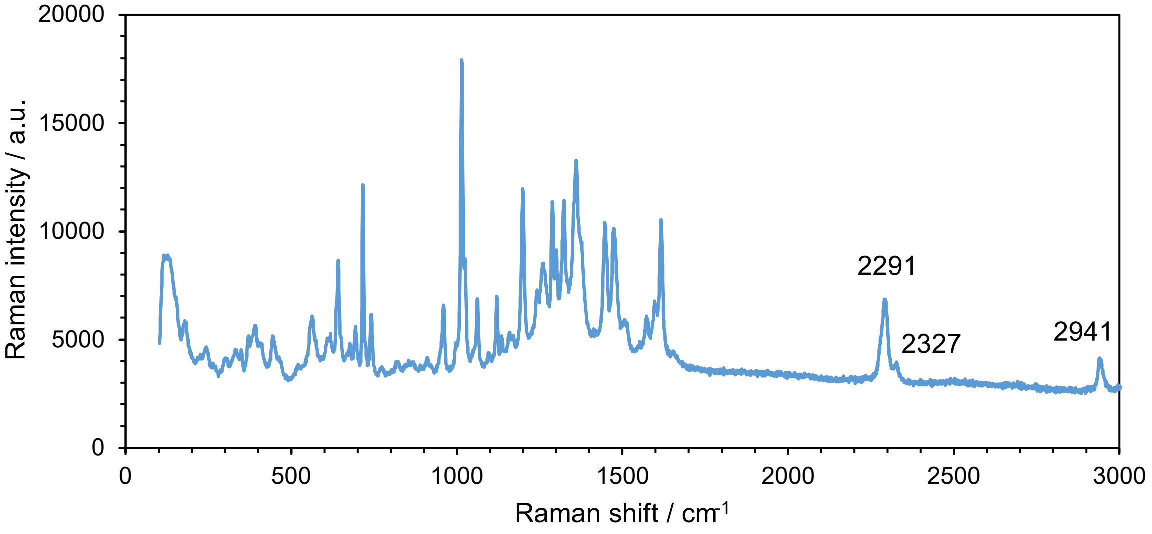

Figure 9 SI. Raman spectrum of **1**. The signals at 2291, 2327 and 2941 cm^{-1} can be assigned to MeCN vibrations according to the literature.(Zhang *et al.*, 2021)

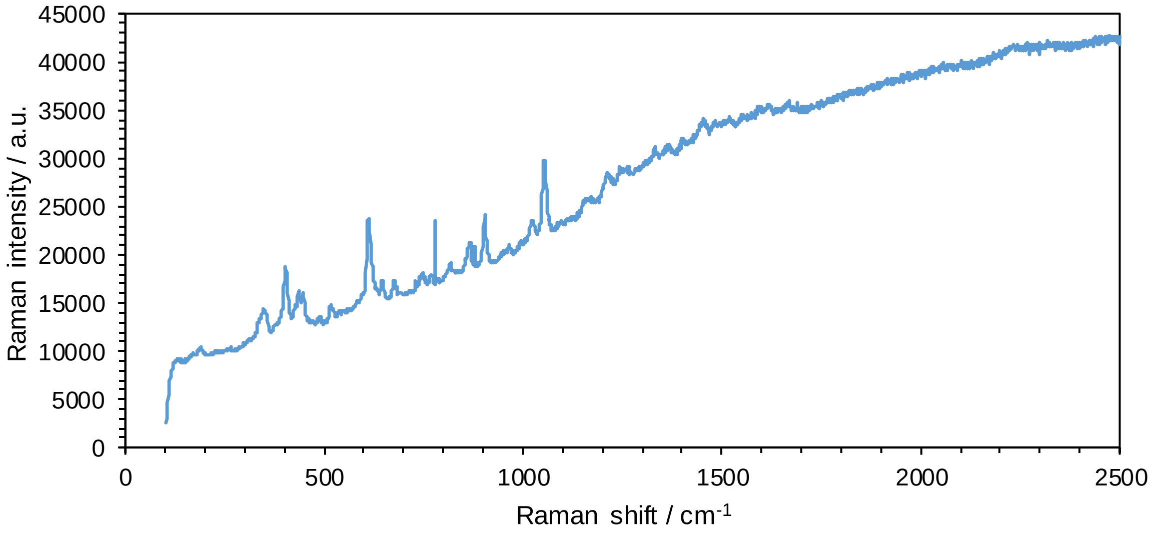

Figure 10 SI. Raman spectrum of crystalline crude material of the crystallization batch of **2**. No NN band is visible.

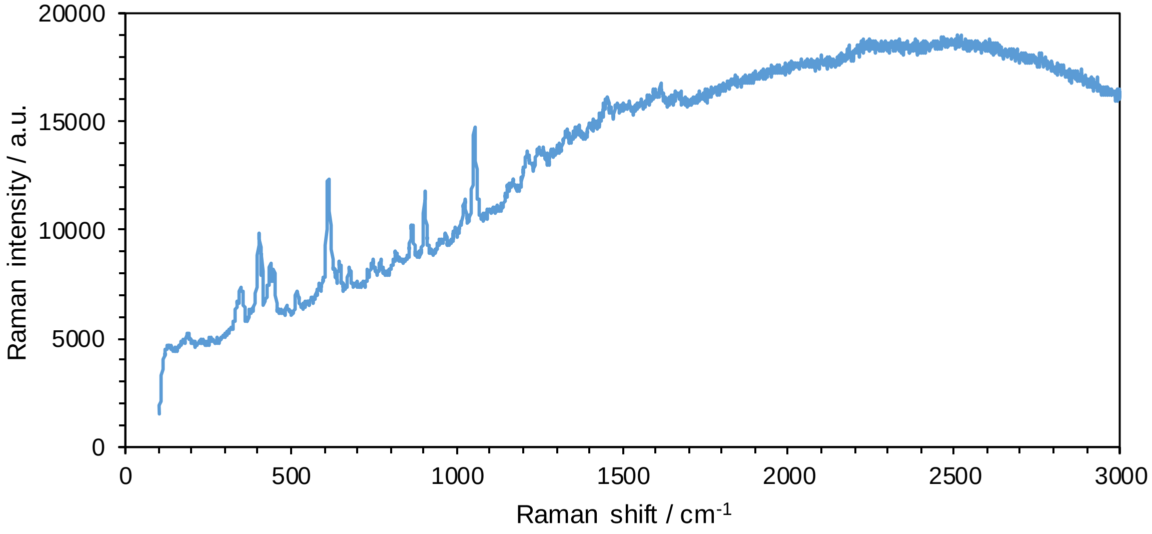

Figure 11 SI. Raman spectrum of crude material of the crystallization batch of **2**. No NN band is visible.

5. References SI

Armarego, W. L. (2017). *Purification of laboratory chemicals*. Butterworth-Heinemann.

Deng, D., Gopiraman, M., Kim, S. H., Chung, I.-M. & Kim, I. S. (2016). *ACS Sustainable Chemistry & Engineering* **4**, 5409-5414.

Dereka, B., Lewis, N. H. C., Keim, J. H., Snyder, S. A. & Tokmakoff, A. (2022). *J. Phys. Chem. B* **126**, 278-291.

Fulmer, G. R., Miller, A. J. M., Sherden, N. H., Gottlieb, H. E., Nudelman, A., Stoltz, B. M., Bercaw, J. E. & Goldberg, K. I. (2010). *Organometallics* **29**, 2176-2179.

Haveli, S. D., Walter, P., Patriarche, G., Ayache, J., Castaing, J., Van Elslande, E., Tsoucaris, G., Wang, P.-A. & Kagan, H. B. (2012). *Nano Lett.* **12**, 6212-6217.

Liu, X., Zhou, W., Yang, L., Li, L., Zhang, Z., Ke, Y. & Chen, S. (2015). *Journal of Materials Chemistry A* **3**, 8840-8846.

Pruppacher, H. R. & Klett, J. D. (1997). *Microphysics of Clouds and Precipitation*. Springer Dordrecht.

Sear, R. P. (2014). *CrystEngComm* **16**, 6506-6522.

Walter, P., Welcomme, E., Hallégot, P., Zaluzec, N. J., Deeb, C., Castaing, J., Veyssière, P., Bréniaux, R., Lévêque, J.-L. & Tsoucaris, G. (2006). *Nano Lett.* **6**, 2215-2219.

Zhang, S., Jia, H., Song, M., Shen, H., Dongfei, L. & Haibo, L. (2021). *Spectrochim. Acta A Mol. Biomol. Spectrosc.* **246**, 119065.
